# Analysis of seed-associated bacteria and fungi on staple crops using the cultivation and metagenomic approaches

**DOI:** 10.1007/s12223-022-00958-5

**Published:** 2022-02-26

**Authors:** Valerija Tkalec, Aleksander Mahnic, Peter Gselman, Maja Rupnik

**Affiliations:** 1 Department for Microbiological Research, National Laboratory for Health, Environment and Food, Maribor, Slovenia; 2 Department of Microbiology, Faculty of Medicine, University of Maribor, Maribor, Slovenia; 3 INTERKORN d.o.o, Beltinci, Slovenia

**Keywords:** Microbiota, Wheat, Barley, Corn, NGS, Culture

## Abstract

One of the key factors affecting seed quality is microbial communities residing on and in the seeds. In this study, microbial populations of seeds of conventionally and organically produced wheat, barley, and maize were analyzed using two different approaches: the cultivation method and metagenomics. For cultivation, three basic media were used: DG18 (for fungi), and nutrient agar or tryptic soy agar supplemented with cycloheximide or nystatin (for bacteria). Metagenomic sequencing was performed using the Illumina MiSeq platform. A total of 452 bacterial isolates comprising 36 genera and 5 phyla and 90 fungal isolates comprising 10 genera and 3 phyla were obtained from the seed surfaces. Among bacteria, representatives from the genera *Bacillus*, *Pantoea*, *Paenibacillus*, and *Curtobacterium* predominated, and among fungi, *Aspergillus* predominated. A total of 142 fungal OTUs and 201 bacterial OTUs were obtained from all the samples. Proteobacteria, Firmicutes, Bacteroides, and Actinobacteria comprised most of the bacterial OTUs, and Ascomycota comprised most of the fungal OTUs. Only 3 fungal OTUs (representatives of *Curvibasidium*, *Venturia*, and *Dermateaceae*) were exclusively present only within seeds and not on the seed surfaces. Barley seeds had the highest microbial load and richness, whereas corn had the lowest. Wheat and barley shared a higher number of OTUs than either of them did with corn with higher overlap between conventionally grown cereals than between organically grown cereals. Some OTUs were farming specific. This study demonstrates that the microbiome of cereal seeds is greatly dependent on the species of the host and is less affected by agricultural practices.

**Supplementary Information:**

The online version contains supplementary material available at 10.1007/s12223-022-00958-5.

## Introduction

In 2019, more than 299 million tons of cereal were produced in the European Union, with maize, wheat, rice, and barley being the four most extensively grown cereals (Eurostat 2020). Their continuous massive production is enabled by high-quality seeds. Seed quality is strongly affected by microbial communities residing in internal seed tissues (i.e., endophytes) and on seed surfaces (i.e., epiphytes). Seed-associated microbial populations are implicated in germination, plant growth and development, and seed storage. The structure of seed-associated microbial communities is driven by plant genotype, the environment, and management practices (Berg and Raaijmakers 2018).

Seed-associated microbial populations are composed of up to 9000 different synergistic, commensal, and potentially pathogenic microbial species (Berg and Raaijmakers 2018; Mekonnen 2020). The population size of seeds has generally been determined using cultivation methods. Studies have reported numbers of bacterial endophytes ranging from 10^2^ (Compant et al. 2011; Ferreira et al. 2008; Rosenblueth et al. 2012) to 10^6^–10^8^ bacterial epiphyte CFU/g of seeds (Mano et al. 2006). The most common bacterial endophytes are representatives of the phyla Proteobacteria (e.g., *Pantoea*, *Pseudomonas*, *Massilia*, *Xanthomonas*, and *Telluria* (Links et al. 2014)), Actinobacteria, Firmicutes, and Bacteroidetes (Barret et al. 2015; Bulgarelli et al. 2013; Johnston-Monje et al. 2016; Liu et al. 2012). It was shown that seed-associated bacteria can improve plant growth in suboptimal growth conditions, especially enhancing plant tolerance to heavy metal stress (Gagne-Bourgue et al. 2013; Truyens et al. 2014; Li et al. 2019). Several endophytes isolated from seeds have been shown to possess anti-fungal properties, revealing their potential as biocontrol agents in agriculture (Truyens et al. 2014; Li et al. 2019).

Seeds are also associated with many Ascomycota and Basidiomycota fungi and yeast from the classes Dothideomycetes, Eurotiomycetes, Leotiomycetes, Sordariomycetes, and Tremellomycetes. The class Dothideomycetes includes several important genera, including *Alternaria*, *Aureobasidium*, *Cladosporium*, *Epicoccum*, *Phaeosphaeria*, *Phoma*, *Pyrenophora*, and *Stagonospora.* Other common representatives from Ascomycota include *Chaetomium*, *Fusarium* (and associated teleomorphs), *Microdochium*, *Stemphylium*, and *Xylaria*. Similarly to bacteria, also mycobionts are beneficial to the host, as they enhance seed germination and plant growth (Li et al. 2019).

In contrast to seed tissue-associated endophytes, seed surface-associated epiphytes have been much less studied; especially their role in plant development and health is poorly understood. According to Links et al. (2014), endophytic bacterial communities are rather unique to plant genera, whereas epiphytic bacterial communities are more widespread across different plant genera. Epiphytic fungal communities of cereal seeds are reported to be dominated by the genera *Fusarium*, *Phoma*, *Pyrenophora*, *Alternaria*, and *Leptosphaeria*, which include well-known plant pathogens (Links et al. 2014; Rahman et al. 2016; Nelson 2018; Nelson et al. 2018; Xing et al. 2018; Solanki et al. 2019).

In the past decade, the number of studies on seed-associated microbiomes has been increasing. Because of the implementation of culturing data with the sequence analysis approach, much deeper insight into the taxonomic diversity of seed microbiota is now available (Links et al. 2014). A more extensive understanding of seed microbiota could contribute to better manipulation strategies of the plant microbiome and better protection against abiotic and biotic factors, thus enhancing plant growth and production yield. The objective of the present study was to characterize the bacteria and fungi present on maize, wheat, and barley seeds produced by two different farming systems, organic and conventional using two different approaches: cultivation and sequencing.

## Materials and methods

### Seed samples

Six samples were collected, comprising seeds of wheat (*n*=2), barley (*n*=2), and corn (*n*=2), each of which was produced in conventional and organic farming systems. Wheat was harvested in June 2018, barley in July 2018, and corn in October 2018 in the Eastern region of Slovenia. The grains were deposited in paper bags (2 kg per bag) and stored for 6 months at standard storage conditions (20 °C, 50% humidity).

### Cultivation and identification of isolates

Two cultivation approaches were used in this study: (1) direct cultivation of seeds on selected media to identify cultivable bacteria and fungi and (2) cultivation of grain rinse to evaluate the microbial burden on seeds based on the colony-forming unit (CFU) count. However, this method could not determine the fungal CFU count and was thus only used to determine bacterial CFU count. Both approaches are described below in more detail. Altogether, five different media were used. All were prepared in-house according to the manufacturer’s instructions. Fungi were cultivated on DG18 (Biolife) supplemented with chloramphenicol (100 mg/L; Biolife), and bacteria were cultivated on nutrient agar (Biolife) or tryptic soy agar (Oxoid) supplemented with cycloheximide (25 mg/L; Biolife) or nystatin (25 mg/L; Biolife).

### Direct cultivation from seeds

For direct inoculation, seeds (*n*=1–5 seeds per plate) were placed onto the five different media described above, and the plates were shaken in all directions for 30 s. The number of plated seeds differed according to the microbial burden determined in the preliminary experiments (data not shown). For barley, only one seed per plate was used, regardless of the farming type or microorganism (bacteria vs. fungi) analyzed. For wheat, the number of seeds had to be adjusted according to the farming type (conventional wheat: *n*=1 seed per plate, organic wheat: *n*=3 seeds per plate). For corn, the number of seeds depended on the microorganism analyzed (fungi: *n*=1 seed per plate, bacteria: *n*=5 seeds per plate).

The bacteriological plates (nutrient agar or tryptic soy agar with cycloheximide or nystatin) were incubated for 3–5 days at room temperature. After incubation, up to 27 colonies of different morphologies per single plate were randomly selected, transferred to a fresh plate, and identified by protein mass spectrometry (MALDI-TOF MS; Bruker) or 16S rDNA analysis. Fungi were examined after 3–5 days of incubation at 30 °C. Up to 27 colonies per single plate were transferred to a fresh plate and identified by analyzing the internal transcribed spacer (ITS) region.

The ITS and 16S rDNA were amplified by PCR and underwent Sanger sequencing according to our routine laboratory protocols using the primers 27fevb GAGAGTTTGATCCTGGCTCAG and 1495revb CTACGGCTACCTTGTTACGA (Bianciotto et al. 1996) for 16S rDNA and two pairs of primers for the ITS region: ITS 1/4 and ITS 86/4 (ITS1: TCCGTAGGTGAACCTGCGG, ITS4: TCCTCCGCTTATTGATATGC, according to White et al. (1990), and ITS86F: GTGAATCATCGAATCTTTGAA, published by Op De Beeck et al. (2014). The determined 16S rDNA sequences were compared to sequences available in the GenBank, The Ribosomal Database Project (RDP) II, and MicrobeNet databases, and ITS sequences were compared to sequences in the GenBank and ISHAM databases.

### Evaluation of the bacterial load on seeds

To evaluate the bacterial load on seeds, rinse of the seeds was prepared using vortexing and sonication. First, 1 mL of phosphate-buffered saline was added to 10 seeds of each sample and vortexed for 1 min, followed by sonication for 1 min at 100% power (BactoSonic, Bandelin). Next, 100 μL of undiluted rinse and 10^−1^–10^−5^ dilutions were plated onto tryptic soy agar or nutrient agar with cycloheximide in duplicates. After 3 days of incubation at room temperature, colonies on agar plates were counted and the number of CFU per grain (CFU/grain) determined.

### Sample preparation and DNA extraction for sequencing

Seeds corresponding to a volume of 10 mL were added to 7 mL of phosphate-buffered saline in duplicates. One sample was subjected to vortexing, and additional one to a combination of vortexing (1 min) and sonication (1 min) at 100% power (BactoSonic, Bandelin). Next, 1 mL from each rinse was centrifuged at 12 000 rpm/min for 10 min, and the pellet was used for total DNA isolation. In parallel, for molecular endophyte analysis, 10 mL of sample was treated with 30 mL of 1% bleach for 5 min and washed three times with 30 mL of sterile distilled water. The seeds were then air-dried, transferred to a Stomacher bag (Interscience), and mechanically macerated in a mortar. Altogether, 50 mg of macerated grain content was transferred to a fresh tube and used for DNA isolation.

Total DNA was extracted with the QIAamp DNA Mini Kit (QIAGEN) according to the manufacturer’s protocol but with one additional step. First, the pellets were re-suspended in 250 μL of ATL buffer (a tissue lysis buffer; QIAGEN) and homogenized in SeptiFast tubes (Roche) with MagnaLyser (Roche) at 7000 rpm for 70 s. Altogether, 180 μL of suspension was transferred to a fresh tube, and 20 μL of proteinase K (QIAGEN) was added. The subsequent steps were carried out according to the manufacturer’s protocol. The extracted DNA was stored at −80 °C until further use.

### Amplicon library preparation and sequencing

Bacterial community structure was determined by sequencing the V3V4 variable region of the 16S rRNA gene. Libraries were prepared according to the 16S Metagenomic Sequencing Library Preparation (Illumina) protocol using the primer pair Bakt_341F (5′-CCTACGGGNGGCWGCAG-3′) – Bakt_805R (5′-GACTACHVGGGTATCTAATCC-3′); the average fragment length was 460 bp. The fungal community was determined by sequencing the ITS2 using a broad-range set of primers: ITS86F (5′-GTGAATCATCGAATCTTTGAA-3′) – ITS4R (5′-TCCTCCGCTTATTGATATGC-3′); the average fragment length was 280 bp. The library was again prepared according to the 16S Metagenomic Sequencing Library Preparation manual (Illumina, CA, USA), with the exception that Q5 High-Fidelity DNA Polymerase (NEB, MA, USA) was used instead of KAPA HiFi HotStart ReadyMix (Kapa Biosystems). Library qualities were estimated using the Bioanalyzer High Sensitivity DNA Analysis Kit (Agilent). Sequencing was performed on the Illumina MiSeq (paired-end sequencing, 2×300 bp, 5% PhiX).

### Sequence data analysis and statistics

Quality filtering of the sequence reads was performed in the mothur environment (v.1.36.1) (Schloss et al. 2009), generally following the protocol established by Kozich et al. (2013). V3V4 amplicon reads were aligned using the Silva reference base (Release 123). Chimeras were identified using a mothur-implemented UCHIME algorithm. Taxonomy was inferred with the RDP training set (v.12) (0.80 bootstrap value). Unique reads that were represented in the relative abundance below 0.01% were removed.

ITS2 amplicon reads were first binned using the ITSx tool to remove non-fungal reads (Bengtsson-Palme et al. 2013). Fungal reads were then pairwise aligned using the Needleman-Wunsch method. Taxonomy was inferred using the UNITE ITS database (version 6) with a bootstrap threshold value of 0.80. Unique reads that were represented in the relative abundance below 0.01% were removed.

Four negative controls were included in the experiment, following each step from the sample preparation onwards. To minimize the possibility of any false-positive detections, we implemented the following algorithm to remove potential contaminants from the microbial communities. For each OTU, we determined the threshold, which was calculated as 5 × *N*_max_, where *N*_max_ is equal to the read count in the negative control with the largest number of reads for the respective OTU. The respective OTU was then removed from the samples where the read count was lower than the threshold. Finally, we obtained a total of 562 070 bacterial reads (min: 856, max: 103 854, average per sample: 26 765.2) and 1 398 644 fungal reads (min: 1, max: 199 190, average per sample: 66 602.1). Statistical analysis was performed in the mothur environment (alpha diversity) and in R using the package “ggplot2.”

## Results

The bacterial and fungal communities on the seed surfaces of wheat, barley, and corn were analyzed using molecular approaches and cultivation. For molecular detection, two protocols for sample preparation were used: (a) a simple rinse obtained by vortexing and (b) vortexing followed by sonication. The sonication step increased the number of detected OTUs in some samples (Tables S1, S2 and S3). The final analysis was done on all the OTUs determined from both protocols, unless stated otherwise. The sonicated rinse was also used to evaluate the bacterial overall burden by cultivation. However, to increase the diversity of the obtained cultivable bacteria and fungi, another cultivation approach, direct grain plating, was employed. In addition, surface-sterilized macerated grains were included in the molecular analysis to detect bacteria and fungi residing inside the grains.

### Bacterial and fungal populations of all tested cereals determined by cultivation and amplicon sequencing

By cultivation, a total of 452 bacterial isolates comprising 36 genera and 5 phyla (Table 1) and 90 fungal isolates comprising 10 genera and 3 phyla (Table 2) were obtained. Representatives from the genera *Bacillus*, *Pantoea*, *Paenibacillus*, and *Curtobacterium* predominated (70.4% of all isolates) among the bacterial isolates. Representatives from *Aspergillus* followed by *Alternaria*, *Penicillium*, and *Fusarium* predominated (78.9% of all isolates) among the fungal isolates (Tables 1 and 2).

**Table 1:** The bacterial isolates obtained from different cereals and farming systems

**Genera**	**Nr. of determined species per specified genus**	**Number of isolates**						**Total Nr. of isolates per specified genus**
**Cereal**		**Wheat**		**Barley**		**Corn**		
**Farming system**		**Conventional**	**Organic**	**Conventional**	**Organic**	**Conventional**	**Organic**	
**Bacterial burden (CFU/grain)**	na	7.1×10^4^	4.5	4.3×10^4^	6.6×10^4^	5.4	3.6	na
*Bacillus*	21	28	14	31	32	16	5	126
*Pantoea*	1^a^	35	2	24	24	-	-	85
*Paenibacillus*	6^b^	14	2	18	13	3	5	55
*Curtobacterium*	3	17	10	11	8	4	2	52
*Pseudomonas*	10	11	-	9	18	-	-	38
*Microbacterium*	5^c^	5	-	12	8	1	-	26
*Kosakonia*	1	7	-	8	1	-	-	16
*Rhodococcus*	1	1	-	4	3	-	-	8
*Pseudoclavibacter*	1	-	-	3	3	1	-	7
*Exiguobacterium*	/	-	-	3	2	-	-	5
*Staphylococcus*	1	-	-	2	1	-	-	3
*Cronobacter*	/	-	-	1	1	-	-	2
*Lysinibacillus*	2	1	-	-	1	-	-	2
*Plantibacter*	1	-	-	2	-	-	-	2
*Rathayibacter*	/	-	1	1	-	-	-	2
*Saccharibacillus*	/	-	-	1	1	-	-	2
*Stenostrophomonas*	2	-	-	2	-	-	-	2
*Acinetobacter*	/	**-**	**-**	**-**	1	-	-	1
*Agrococcus*	1	**-**	**-**	**-**	1	-	-	1
*Arthrobacter*	1	**-**	**-**	1	-	-	-	1
*Brevibacillus*	1	-	1	-	-	-	-	1
*Brevundimonas*	1	-	-	1	-	-	-	1
*Chryseobacterium*	1	-	-	-	1	-	-	1
*Clavibacter*	1	1	-	-	-	-	-	1
*Enterobacter*	1	-	-	-	1	-	-	1
*Escherichia*	1	-	-	1	-	-	-	1
*Gordonia*	1	1	-	-	-	-	-	1
*Kocuria*	1	-	-	1	-	-	-	1
*Massilia*	1	-	-	1	-	-	-	1
*Micrococcus*	1	1	-	-	-	-	-	1
*Rummeliibacillus*	1	-	1	-	-	-	-	1
*Sanguibacter*	1	-	-	1	-	-	-	1
*Solibacillus*	1	-	-	1	-	-	-	1
*Sporosarcina*	1	-	1	-	-	-	-	1
*Streptococcus*	1	-	-	-	-	1	-	1
*Viridibacillus*	1	1	-	-	-	-	-	1
Total Nr.	73	123	32	139	120	26	12	452

/, identified only to the genus level; -, zero isolates obtained

^a–c^Isolates identified only to the genus level (^a^*n*=5, ^b^*n*=5, ^c^*n*=10)

**Table 2:** The fungal isolates obtained from different cereals and farming systems

**Genera**	**Nr. of determined species per specified genus**	**Number of isolates**						**Total Nr. of isolates per specified genus**
**Cereal**		**Wheat**		**Barley**		**Corn**		
**Farming system**		**Conventional**	**Organic**	**Conventional**	**Organic**	**Conventional**	**Organic**	
*Alternaria*	2	5	-	7	8	-	-	20
*Arthrinium*	1	1	-	-	-	-	-	1
*Aspergillus*	5^a^	12	4	3	2	2	2	25
*Aureobasidium*	1	-	-	1	2	-	-	3
*Cladosporium*	2^b^	2	-	3	2	-	-	7
*Fusarium*	3^c^	-	-	4	5	-	3	12
*Mucor*	1	-	-	2	-	-	-	2
*Penicillium*	2^d^	-	1	6	4	1	2	14
*Rhizopus*	/	-	-	-	-	1	1	2
*Trichoderma*	1^e^	-	4	-	-	-	-	4
Total Nr.	18	**20**	**9**	**26**	**23**	**4**	**8**	90

/, identified only to the genus level; -, zero isolates obtained

^a–e^Isolates identified only to the genus level (^a^*n*=13, ^b^*n*=3, ^c^*n*=7, ^d^*n*=8, ^e^*n*=1)

After quality filtering and the removal of low abundant reads and potential contaminants from the NGS sequence analysis, a total of 142 fungal OTUs and 201 bacterial OTUs were obtained from all the studied samples (Tables S1 and S2).

**Fig. 1:**
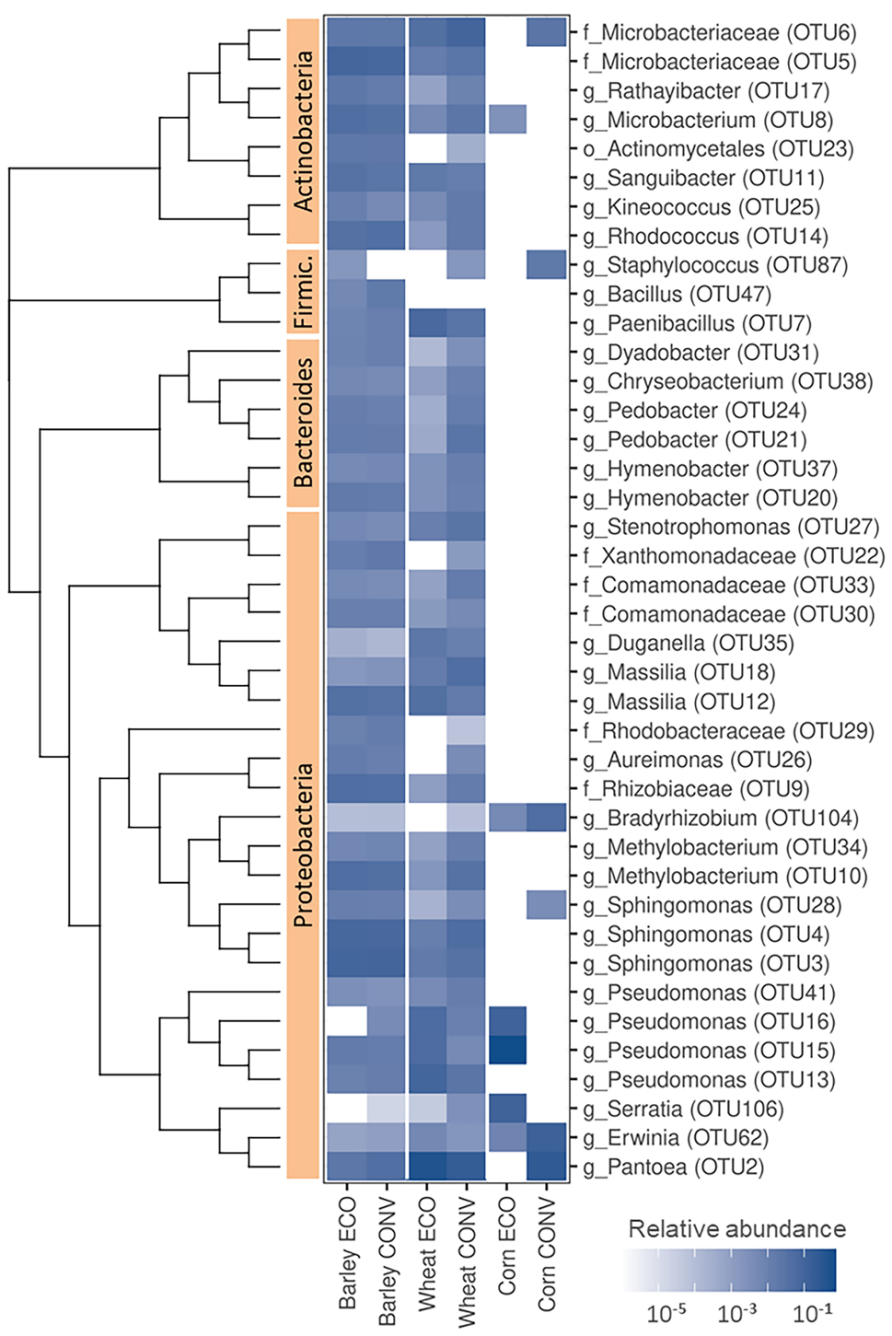
The most abundant bacterial groups detected by 16S-metagenomics on the surfaces of different seeds. The heat plot presents the relative abundances of bacterial OTUs for barley, wheat, and corn grown either organically (ECO) or conventionally (CONV). Letters before the underscores denote the top taxo*-nomical level that could be assigned to the OTU; g, genus; f, family; o, order. Phylogeny is presented as a neighbor-joining tree constructed from Bray-Curtis dissimilarities between OTU representative sequences

**Fig. 2:**
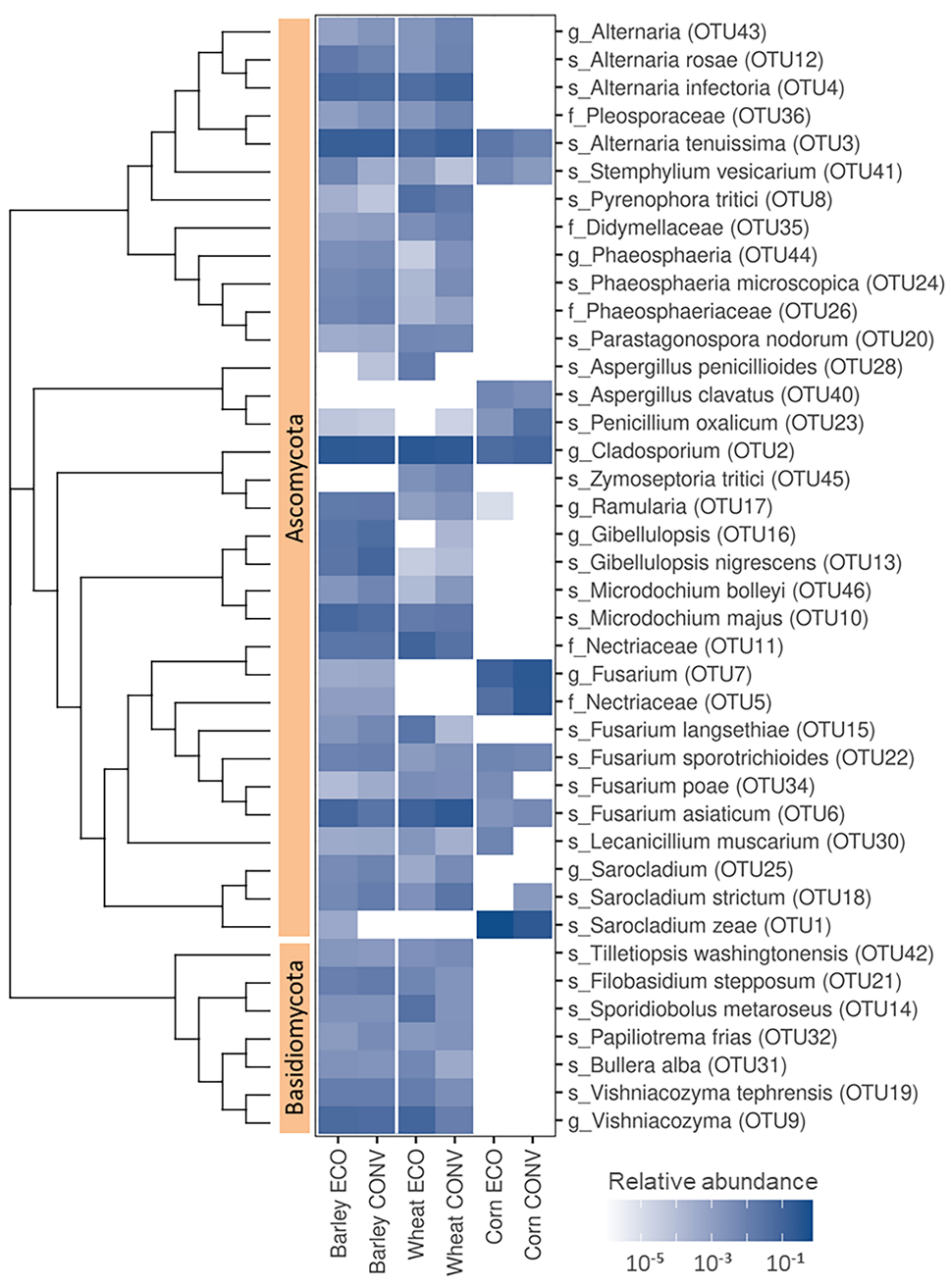
The most abundant fungal groups found on the surfaces of different seeds. The heat plot presents the relative abundances of fungal OTUs for barley, wheat, and corn produced either organically (ECO) or conventionally (CONV). Letters before the underscores denote the highest taxonomical level that could be assigned to the OTU; s, species; g, genus; f, family. Phylogeny is presented as a neighbor-joining tree constructed from Bray-Curtis dissimilarities between OTU representative sequences

The bacterial communities detected on the seed surfaces were diverse, mainly comprising representatives from the phyla Proteobacteria, Firmicutes, Bacteroides, and Actinobacteria (*n*=168 OTUs; Fig. 1), while Acidobacteria, Gemmatimonadetes, Verrucomicrobia, Nitrospirae, Planctomycetes, and candidate division_WPS-1, *Candidatus Saccharibacteria* (*n*=33 OTUs) were less common (Fig. 1, Table S1). The fungal communities were dominated by representatives from the phylum Ascomycota (*n*=109 OTUs; Fig. 2). The remainder of the fungal communities comprised representatives from the phylum Basidiomycota (*n*=33 OTUs) (Fig. 2, Table S2).

While 201 bacterial and 140 fungal OTUs were found on the seed surfaces, 101 bacterial and 109 fungal OTUs were found in the macerated samples (Tables S1 and S2). However, from these, only three fungal OTUs (*Curvibasidium*, *Venturia*, and a representative of the family Dermateaceae) were not detected in any surface microbial community. An additional 17 fungal OTUs and 13 bacterial OTUs were detected in the macerated samples that had a relative abundance more than 10-fold greater than that on the seed surfaces (Tables S1 and S2).

### Wheat microbiota determined by cultivation and molecular approaches

The bacterial burden, determined as CFU/grain, was 7.1×10^4^ CFU/grain for conventionally grown wheat and 4.5 CFU/grain (significantly less; *p*<0.001) for organically grown wheat (Table 1). In total, 155 bacterial isolates were cultivated from wheat, 123 (79.4%) from conventional farming and 32 (20.6%) from organic farming (Table 1). Isolates obtained from conventionally and organically produced wheat comprised 13 and 8 genera, respectively. *Bacillus*, *Pantoea*, *Paenibacillus*, and *Curtobacterium* predominated in both types, where only one species per genus (as in the example of *Pantoea*) to up to 10 determined species per genus were determined (e.g., *Bacillus*). In conventionally produced wheat, representatives of *Pseudomonas*, *Microbacterium*, and *Kosakonia* were also frequently isolated (Tables 1 and S4). In total, 29 fungal isolates were obtained from wheat, comprising 6 genera. Only one genus, *Aspergillus*, was common to both wheat types and represented the majority (16/29; 55.2%) of the recovered isolates (Tables 2 and S5).

A total of 149 bacterial OTUs were determined from conventionally produced wheat and 87 from organically produced wheat. All bacterial OTUs (*n*=153) from wheat were distributed into 8 phyla and 62 genera. Of these, 44 OTUs could not be classified at the genus level, and 10 out of these 44 OTUs could only be classified at the phylum level (Table S1). Representatives from the phylum Proteobacteria predominated, with *Pantoea* being the most abundant genus in both wheat types, followed by representatives of Microbacteriaceae and *Sphingomonas* in conventionally produced wheat and Paenibacillaceae, Pseudomonadaceae, and Microbacteriaceae in organically produced wheat (Fig. 1).

Comparable to the bacterial community, the fungal community was richer in conventionally produced wheat (*n*=124 OTUs) when compared to organically grown wheat (*n*=106 OTUs). Two phyla were determined, Ascomycota and Basidiomycota, and 48 genera (20 OTUs) could not be classified at the genus level (Table S2). In both wheat types, representatives of Ascomycota predominated (Fig. 2). Considerable overlap of common genera between the wheat production types was observed, with *Cladosporium* prevailing in both (Table S6).

The analysis of surface-sterilized macerated wheat samples from conventional and organic farming resulted in 49 and 9 bacterial OTUs and 40 and 20 fungal OTUs, respectively. All the detected OTUs were also detected in the surface microbial communities (Tables S1 and S2). However, the relative abundances of 7 OTUs were more than 10-fold higher in macerated samples than in surface samples. Four of these differentially represented OTUs were found in organically produced wheat and three in conventionally produced wheat.

### Barley microbiota determined by cultivation and molecular approaches

The bacterial load of barley was comparable to conventionally grown wheat with no significant differences between conventional and organic farming. Altogether, 4.3×10^4^ CFU/grain and 6.6×10^4^ CFU/grain were determined for conventionally and organically produced barley, respectively (Table 1).

The cultivation of bacteria from barley yielded 259 isolates. Altogether, the 139 and 120 isolates recovered from conventionally and organically produced barley comprised 23 and 18 genera, respectively. Similarly to conventionally produced wheat, representatives from *Bacillus*, *Pantoea*, *Paenibacillus*, *Curtobacterium*, *Pseudomonas*, and *Microbacterium* predominated in both types of barley, from which one (e.g., *Kosakonia* or *Pantoea*) to up to 14 species (e.g., *Bacillus*) could be determined within each genus (Tables 1 and S4). A total of 49 fungal isolates from 7 genera were obtained from barley, the majority of which belonged to the genera *Alternaria*, *Penicillium*, and *Fusarium* (Tables 2 and S5).

NGS revealed no differences in the richness or community structure between conventionally and organically produced barley (195 vs. 166 determined bacterial OTUs and 122 vs. 124 fungal OTUs, respectively). Both the bacterial and fungal community structures were very similar to conventionally produced wheat (Tables S1, S2, and S6). *Sphingomonas* and representatives of Microbacteriaceae predominated among bacteria (Fig. 1), while representatives from the genera *Cladosporium* and *Alternaria* predominated among fungi (Fig. 2).

In conventionally produced barley, one fungal OTU was present in the macerated fraction (a representative from Dermateaceae (OTU039)) but was not detected in the surface sample (Table S2). An additional 10 OTUs were more than 10-fold higher in the macerated fractions compared to the surface samples. The relative abundances of OTU020 (*Parastagonospora nodorum*), OTU057 (*Stagonospora* sp.), and OTU051 (*Muriphaeosphaeria* sp.) in the macerated fractions were more than 100-fold higher compared to the surface samples.

### Corn microbiota determined by cultivation and molecular approaches

The bacterial load on corn was low, with only 5.4 CFU/grain and 3.6 CFU/grain detected on conventionally and organically grown corn, respectively (Table 1). Only 38 bacterial isolates were recovered: 26 and 12 isolates from conventionally and organically produced corn, respectively. The isolates comprised six different genera with *Bacillus*, *Paenibacillus*, and *Curtobacterium* predominating in both types of corn. Up to four different species within each genus were determined (Tables 1 and S4).

Furthermore, 12 fungal isolates were recovered from corn: 4 and 8 isolates from conventionally and organically produced corn, respectively. The isolates comprised the genera *Aspergillus*, *Fusarium*, *Penicillium*, and *Rhizopus* (Tables 2 and S5). All of them, except for *Fusarium* (obtained only from organically cultivated corn), were isolated from both corn types.

Sequencing of corn rinse resulted in a low number of reads. Only 21 and 20 fungal OTUs were determined for organically and conventionally produced corn, respectively (Table S2). Additionally, only nine bacterial OTUs were determined for each corn type (Table S1). No significant cultivation type-associated differences in richness were observed for corn. *Pantoea* predominated among bacteria (Fig. 1), and *Fusarium*, *Sarocladium*, and *Cladosporium* predominated among fungi (Fig. 2). Two OTUs were detected only in the macerated fractions: *Venturia* sp. (OTU94) in organically grown corn and *Curvibasidium* sp. (OTU102) in conventionally grown corn (Table S2).

### Comparison of bacterial populations between different cereals and production types

The number of cultivated bacterial isolates and NGS reads was associated with the bacterial burden on the seeds. NGS revealed that the bacterial richness was the highest on the surface of barley (average=180.5 OTUs per sample), followed by wheat (average=118.0 OTUs per sample), and significantly lower (*p* < 0.001) on corn (average=9.5 OTUs per sample) (Table S1). In agreement with NGS, most of the different isolates were obtained from barley (259 out of total 452; 57.3%; 139 from conventional and 120 from organic farming), followed by wheat (155 out of total 452; 34.3%; with a significantly higher (*p* < 0.001) bacterial burden on conventionally (*n*=123 isolates) compared to organically (*n*=32 isolates) produced wheat). Corn represented only 8.4% of all the cultivable bacterial isolates obtained in this study (Tables 1 and S4).

**Fig. 3:**
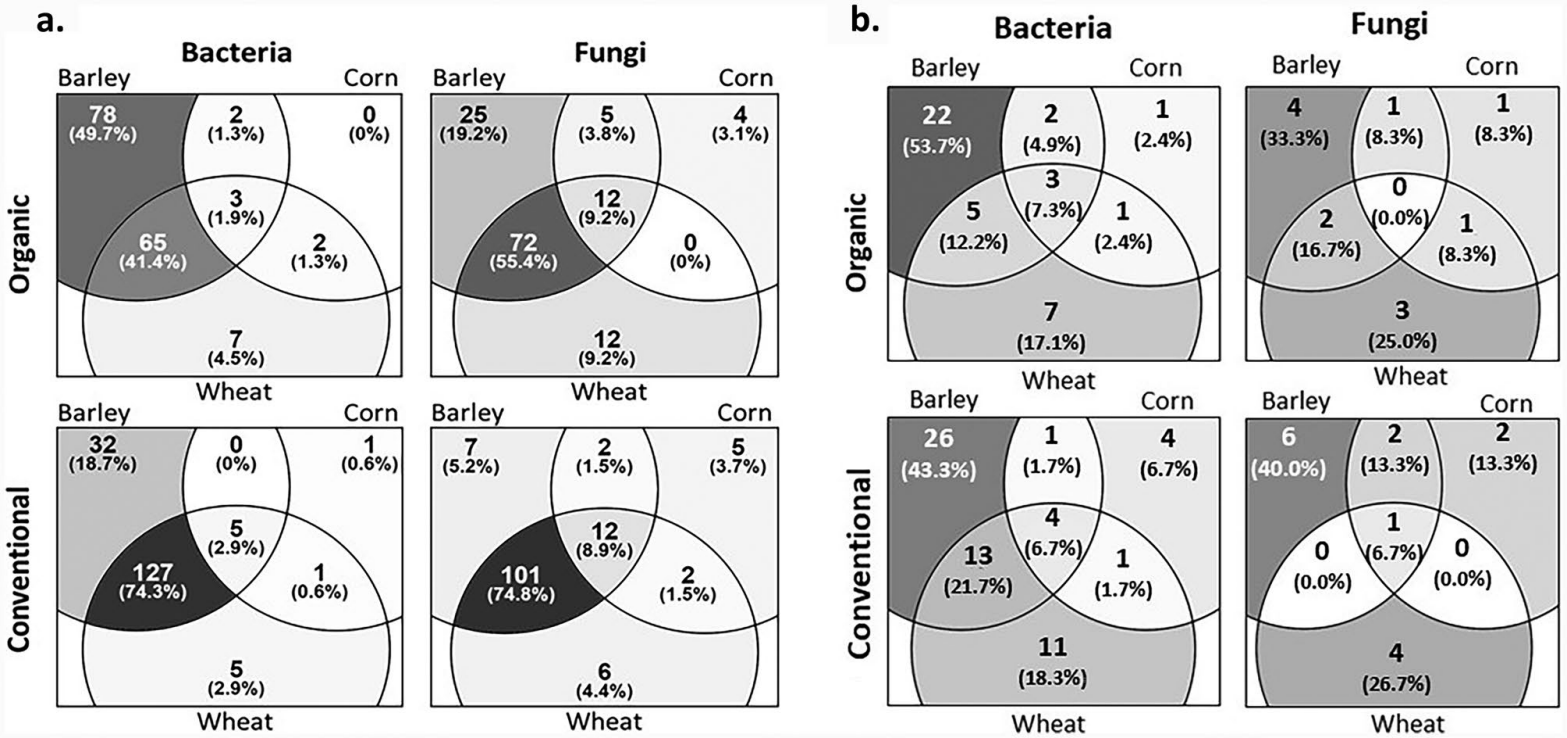
Shared bacterial and fungal species between different seeds. The Venn diagrams show the number and percentage of shared OTUs between barley, corn, and wheat (**a**) and the overlap of cultivated bacterial and fungal species (**b**)

Wheat and barley shared a higher number of bacterial OTUs (> 44.3% of all detected OTUs) than they did with corn (< 3.5%; Fig. 3a). A higher overlap was also observed among conventionally (77.2%) than among organically (43.3%) produced cereals (Fisher’s exact test, *p* < 0.001, Fig. 3a). Similar results were observed with the cultivation method (Fig. 3b).

Although there was generally a considerable overlap of bacterial representatives between conventionally and organically produced seeds, several farming-specific OTUs were determined (Table S6). Overall, one bacterial OTU determined as the genus *Ochrobactrum* was found only in organically produced cereals, and 30 bacterial OTUs comprising nine phyla (Acidobacteria, Firmicutes, Gemmatimonadetes, Proteobacteria, Verrucomicrobia, Bacteroidetes, Planctomycetes, Nitrospirae, candidate_division_WPS-1(100)) were found only in conventionally produced cereals (Tables S1 and S6).

Among all the tested cereals, barley had the highest overlap of bacterial OTUs between conventionally and organically produced seeds (Table S6). Corn had the highest proportion of farming-specific OTUs (43.8% and 37.5% of all bacterial OTUs were found only in conventionally and organically produced corn, respectively), followed by wheat (Table S6).

### Comparison of fungal populations between different cereals and production types

Comparable to bacterial communities, the richness of the fungal communities was significantly higher on the surface of barley and wheat compared to corn (*p* < 0.001), while it did not differ between barley and wheat (Fig. 2). On average, we detected 9.5 OTUs on the surface of corn and 104.3 OTUs on the surface of barley and wheat.

The fungal communities detected on barley and wheat highly overlapped, more so in the case of conventional farming than organic farming (Fisher’s exact test, *p* < 0.001; Fig. 3a). Five fungal groups were found on all six types of seed (three different kinds of cereal, each produced either organically or conventionally), including *Cladosporium* (OTU2), *Alternaria tenuissima* (OTU3), *Fusarium asiaticum* (OTU 6), *Fusarium sporotrichioides* (OTU 22), and *Stemphylium vesicarium* (OTU 41). No significant overlap between cultured fungal isolates from different cereals and production types was determined (Fig. 3b).

Comparing conventional and organic farming, four fungal OTUs were found only in organically produced cereals (Table S6): *Wallemia muriae* (*n*=3) and the genus *Venturia* (*n*=1). Altogether, four fungal OTUs were found only in conventionally produced cereals (Table S6): Ascomycota (the representative could only be determined at the phylum level), *Curvibasidium*, *Acremonium fusidioides*, and *Ceriporiopsis gilvescens.*

Barley had the highest overlap of fungal OTUs between conventionally and organically produced seeds. Corn had the highest proportion of farming-specific OTUs (19.5% and 39.0% of all fungal OTUs were determined only in conventionally and only in organically produced corn, respectively).

## Discussion

In this study, altogether 201 bacterial OTUs and 142 fungal OTUs were obtained by NGS from all six tested samples. A total of 452 bacterial isolates and 90 fugal isolates were obtained by culturing of tested samples and could be differentiated into 73 and 18 species, respectively. As in a study of Amrane et al. (2018) addressing metagenomics and culturonomics, more extensive detection of microbial community representatives was obtained by the NGS method than by culturing also in our study, even though we used different media. However, the unidentified OTUs of still unknown microbial communities (e.g., I 00093: candidate_division_WPS-1(100) from conventionally produced barley (Table S1)) represent one of the disadvantages of NGS. Furthermore, it does not enable the characterization of the detected microorganisms and their functional role within the microbial community. In addition, we observed an under-detection of the sporogenic fraction by the metagenomic approach, as reported before by Filippidou et al. (2015). For example, we detected approximately 20 different *Bacillus* species by culturing, while only 6 OTUs designated as the *Bacillus* genus were determined by NGS.

The bacterial and fungal communities determined on the seed surfaces in this study were diverse and comprised taxa already described in previous seed microbiome studies (Nelson 2018). Proteobacteria, Bacteroidetes, and Actinobacteria were the most common among bacteria in tested seeds, and Ascomycota was the most common among fungi. Barley and wheat shared most OTUs, more so in samples from conventional farming, which is congruent with the concept that microbiota composition depends on seed genotype, environmental conditions, and agricultural practices (Nelson et al. 2018; Safin et al. 2018; Shahzad et al. 2018; Abdullaeva et al. 2020; Ozkurt et al. 2020; Saad et al. 2020; Patil and Maheshwari 2021).

Links et al. (2014) showed that endophyte communities are more unique between different seeds than are epiphyte communities. In our study, a large proportion of OTUs from the seed surfaces was also detected in macerated seeds after surface sterilization. Endophytes can also enter the seed from the environment, and thus an overlap of surface and inner seed OTUs is expected to some extent (Shahzad et al. 2018; Li et al. 2019; Abdullaeva et al. 2020; Ozkurt et al. 2020; Zhou et al. 2020; Patil and Maheshwari 2021; Santos and Olivares 2021). However, we cannot exclude the possibility that the macerated samples were contaminated with surface microbiota in our study, especially those of barley seeds, which have several outer layers that decrease the efficiency of sterilization. As a result, only three fungal OTUs (*Curvibasidium*, *Venturia*, and a representative of the *Dermateaceae* family) were determined as endophytes in our study. An additional 17 fungal and 13 bacterial OTUs were detected that had a more than 10-fold greater relative abundance in the macerated samples than that on the surface of the samples. These were thus designated as potential endophytes.

The microbial burden of seeds was determined by seed rinse analysis. Because the fungal fraction of the microbiome could not be properly rinsed from the seeds, the microbial burden (CFU/grain of tested seeds) was only determined for bacteria. The bacterial burden was the highest in conventionally produced wheat and the lowest in organically produced corn. Interestingly, the burden was comparable between conventionally and organically produced barley and corn, but significantly different between organically and conventionally produced wheat. The difference in the bacterial burden and microbial heterogeneity between organically and conventionally produced wheat seeds could be partially explained by environmental growth conditions, as wheat from organic farming was subjected to notably more drought during its growth and seed development.

Although different farming systems are well established, our understanding of their impact on the microbial diversity of plants and the environment is still not fully understood. Previous studies have focused on the influence of conventional and organic farming systems on the microbial community in soil (Mader et al. 2002; Hartmann et al. 2015; Lupatini et al. 2017; Peltoniemi et al. 2021). These authors reported that organically managed systems increase taxonomic and phylogenetic richness, diversity, and heterogeneity of soil microbiota in comparison with conventional farming systems. However, only few taxonomic groups seemed to consistently correspond to a specific farming system. For example, Proteobacteria and Euryarchaeota have been associated with conventional farming, while Acidobacteria and Planctomycetes have been associated with organic farming (Lupatini et al. 2017).

The different heterogeneities of bacteria and fungi between conventionally and organically produced seeds in our study are not congruent with the results of soil-based studies (Mader et al. 2002; Hartmann et al. 2015; Lupatini et al. 2017; Peltoniemi et al. 2021). In our study, we determined comparable numbers of OTUs between conventionally and organically produced barley and corn. Conversely, we determined a significantly higher number of OTUs conventionally compared to organically produced wheat. The same trend was observed for the determined bacterial burden. However, the observed differences in microbial communities between organically and conventionally produced wheat seeds from our study cannot be generalized due to the small sample size. Indeed, our subsequent studies indicate that microbial heterogeneity and burden between conventionally and organically produced seeds are similar also in wheat (data not shown). A considerable overlap of bacterial and fungal representatives was observed between conventionally and organically produced seeds. Only five OTUs were specific to organically grown seeds (1 bacterial and 4 fungal OTUs), and 34 OTUs were specific to conventionally grown seeds (30 bacterial and 4 fungal OTUs). This suggests that the seed microbiome is less affected by agricultural practices than the soil microbiome. Furthermore, the seed microbiome greatly depends on the species of the host.

To the best of our knowledge, this is the first study to compare seed-associated endophytic and epiphytic microbiomes of major crops produced by different farming systems. By comparison of three different grain types (wheat, barley, corn), two different production systems (organic and conventional), surface and macerated seeds, and two analytical approaches (culturing and sequencing), we have shown that bacterial and fungal seed associated microbiota is diverse, host species specific, and influenced by farming type. Diversity and richness are higher in wheat and barley compared to corn. Only very low proportion of representatives from surface-associated microbiome are also found in a macerated fraction of the seeds.

## Supplementary Information

Below is the link to the electronic supplementary material.

Supplementary file1 (XLSX 32 KB)

Supplementary file2 (XLSX 28 KB)

Supplementary file3 (DOCX 18 KB)

Supplementary file4 (DOCX 32 KB)

Supplementary file5 (DOCX 22 KB)

Supplementary file6 (DOCX 18 KB)
