# Supplementary material for: Analysis of seed-associated bacteria and fungi on staple crops using the cultivation and metagenomic approaches
Source: Folia Microbiol (Praha). 2022 Feb 26;67(3):351–61. doi: 10.1007/s12223-022-00958-5 (PMC9072454; doi:10.1007/s12223-022-00958-5)
Supplement: Supplementary file 3 — Supplementary file3 (DOCX 18 KB) [file 12223_2022_958_MOESM3_ESM.docx]

**Table S3 Comparison of bacterial and fungal community structure (OTUs) between two sample preparation methods for NGS analysis of different seed types**

|  | Cereal/  farming-system | **All detected OTUs^a^** | **Overlap^b^** | **Rinse only^c^** | **Sonication only^d^** |
| --- | --- | --- | --- | --- | --- |
| **Bacteria** | Barley ORG | 166 | 139 | 17 | 10 |
|  | Barley CONV | 195 | 159 | 31 | 5 |
|  | Corn ECO | 9 | 3 | 2 | 4 |
|  | Corn CONV | 9 | 2 | 3 | 4 |
|  | Wheat ECO | 87 | 50 | 10 | 27 |
|  | Wheat CONV | 149 | 124 | 11 | 14 |
| **Fungi** | Barley ECO | 124 | 111 | 10 | 3 |
|  | Barley CONV | 122 | 74 | 0 | 48 |
|  | Corn ECO | 33 | 18 | 12 | 3 |
|  | Corn CONV | 25 | 14 | 4 | 7 |
|  | Wheat ECO | 106 | 88 | 10 | 8 |
|  | Wheat CONV | 124 | 117 | 1 | 6 |

^a^ all detected OTUs in the analysis (using rinsing and/or sonication); ^b^number of OTUs obtained with rinsing that were also detected by sonication^; c^number of OTUs obtained only with rinsing; ^c^number of OTUs obtained only with sonication
