# Supplementary material for: Analysis of seed-associated bacteria and fungi on staple crops using the cultivation and metagenomic approaches
Source: Folia Microbiol (Praha). 2022 Feb 26;67(3):351–61. doi: 10.1007/s12223-022-00958-5 (PMC9072454; doi:10.1007/s12223-022-00958-5)
Supplement: Supplementary file 4 — Supplementary file4 (DOCX 32 KB) [file 12223_2022_958_MOESM4_ESM.docx]

**Table S4 List of all obtained bacterial isolates**

| **Isolated bacteria** | **Wheat** | | **Barley** | | **Corn** | | **Total Nr. of isolates** |
| --- | --- | --- | --- | --- | --- | --- | --- |
| **Farming system** | **conventional** | **organic** | **conventional** | **organic** | **conventional** | **organic** |  |
| *Acinetobacter sp.* | **-** | **-** | **-** | 1 | - | - | 1 |
| *Agrococcus jejuensis* | **-** | **-** | **-** | 1 | - | - | 1 |
| *Arthrobacter gandavensis* | **-** | **-** | 1 | - | - | - | 1 |
| *Bacillus megaterium* | 7 | 2 | 8 | 7 | 4 | - | 28 |
| *Bacillus simplex* | 4 | - | 3 | 9 | - | 1 | 17 |
| *Bacillus pumilus* | 3 | 2 | 2 | 1 | 3 | 1 | 12 |
| *Bacillus thuringiensis* | 3 | 1 | 3 | 4 | - | - | 11 |
| *Bacillus mycoides* | 2 | 1 | 3 | 3 | - | - | 9 |
| *Bacillus subtilis* | 3 | 3 | - | 1 | 2 | - | 9 |
| *Bacillus cereus* | 2 | - | 2 | 3 | - | - | 7 |
| *Bacillus muralis* | 2 | 1 | 1 | 2 | - | 1 | 7 |
| *Bacillus mojavensis* | - | - | - | - | 6 | - | 6 |
| *Bacillus gibsonii* | - | - | 2 | 1 | - | 2 | 5 |
| *Bacillus weihenstephanensis* | - | 1 | 2 | - | - | - | 3 |
| *Bacillus altitunidis* | 1 | - | 1 | - | - | - | 2 |
| *Bacillus sp.* | - | 2 | - | - | - | - | 2 |
| *Bacillus amyloliquefaciens ssp. plantarum* | - | - | - | - | 1 | - | 1 |
| *Bacillus bataviensis* | - | 1 | - | - | - | - | 1 |
| *Bacillus drentensis* | - | - | 1 | - | - | - | 1 |
| *Bacillus fulva* | - | - | 1 | - | - | - | 1 |
| *Bacillus horneckiae* | - | - | 1 | - | - | - | 1 |
| *Bacillus jeotgali* | 1 | - | - | - | - | - | 1 |
| *Bacillus luciferensis* | - | - | 1 | - | - | - | 1 |
| *Bacillus niacini* | - | - | - | 1 | - | - | 1 |
| *Brevibacillus borstelensis* | - | 1 | - | - | - | - | 1 |
| *Brevundimonas vesicularis* | - | - | 1 | - | - | - | 1 |
| *Chryseobacterium indoltheticum* | - | - | - | 1 | - | - | 1 |
| *Clavibacter michiganensis* | 1 | - | - | - | - | - | 1 |
| *Cronobacter sp.* | - | - | 1 | 1 | - | - | 2 |
| *Curtobacterium flaccumfaciens* | 11 | 5 | 9 | 8 | 4 | 2 | 39 |
| *Curtobacterium albidum* | 3 | 3 | 2 | - | - | - | 8 |
| *Curtobacterium herbarum* | 3 | 2 | - | - | - | - | 5 |
| *Enterobacter asburiae* | - | - | - | 1 | - | - | 1 |
| *Escherichia coli* | - | - | 1 | - | - | - | 1 |
| *Exiguobacterium sp.* | - | - | 3 | 2 | - | - | 5 |
| *Gordonia rubripertincta* | 1 | - | - | - | - | - | 1 |
| *Kocuria rhizophila* | - | - | 1 | - | - | - | 1 |
| *Kosakonia cowanii* | 7 | - | 8 | 1 | - | - | 16 |
| *Lysinibacillus fusiformis* | - | - | - | 1 | - | - | 1 |
| *Lysinibacillus xylanilyticus* | 1 | - | - | - | - | - | 1 |
| *Massilia oculi* | - | - | 1 | - | - | - | 1 |
| *Microbacterium sp./ Okibacterium fritillariae* | - | - | 6 | 3 | - | - | 9 |
| *Microbacterium testaceum* | 5 | - | - | - | 1 |  | 6 |
| *Microbacterium testaceum* | - | - | 2 | 3 | - | - | 5 |
| *Microbacterium foliorum* | - | - | 2 | 1 | - | - | 3 |
| *Microbacterium arborescens* | - | - | 1 | 1 | - | - | 2 |
| *Microbacterium phyllosphaerae* | - | - | 1 | - | - | - | 1 |
| *Micrococcus luteus* | 1 | - | - | - | - | - | 1 |
| *Paenibacillus nicotianae* | 8 | - | 3 | 3 | - | - | 14 |
| *Paenibacillus illinoisensis* | 2 | - | 3 | 8 | - | - | 13 |
| *Paenibacillus amylolyticus* | - | 1 | 8 |  | - | 1 | 10 |
| *Paenibacillus sp.* | 3 | 1 | 3 | 1 | 2 | - | 10 |
| *Paenibacillus barcinonensis* | - | - | 1 | - | - | 4 | 5 |
| *Paenibacillus hordei* | 1 | - | - | 1 | - | - | 2 |
| *Paenibacillus provencensis* | - | - | - | - | 1 |  | 1 |
| *Pantoea agglomerans* | 35 | 2 | 21 | 22 | - | - | 80 |
| *Pantoea sp.* | - | - | 3 | 2 | - | - | 5 |
| *Plantibacter flavus* | - | - | - | 2 | - | - | 2 |
| *Pseudoclavibacter helvolus* | - | - | 3 | 3 | 1 | - | 7 |
| *Pseudomonas oryzihabitans* | 5 | - | 3 | 6 | - | - | 14 |
| *Pseudomonas flavescens* | - | - | 2 | 8 | - | - | 10 |
| *Pseudomonas antarctica* | 3 | - |  | 1 | - | - | 4 |
| *Pseudomonas cichorii* | - | - | 2 | - | - | - | 2 |
| *Pseudomonas poae* | 1 | - |  | 1 | - | - | 2 |
| *Pseudomonas tolaasii* | 2 | - |  | - | - | - | 2 |
| *Pseudomonas fulva* | - | - | 1 | - | - | - | 1 |
| *Pseudomonas libanensis* | - | - | - | 1 | - | - | 1 |
| *Pseudomonas sp.* | - | - | - | 1 | - | - | 1 |
| *Pseudomonas viridiflava* | - | - | 1 | - | - | - | 1 |
| *Rathayibacter sp.* | - | 1 | 1 | - | - | - | 2 |
| *Rhodococcus fascians* | 1 | - | 4 | 3 | - | - | 8 |
| *Rummeliibacillus stabekisii* | - | 1 | - | - | - | - | 1 |
| *Saccharibacillus sp.* | - | - | 1 | 1 | - | - | 2 |
| *Sanguibacter inulinus* | - | - | 1 | - | - | - | 1 |
| *Solibacillus silvestris* | - | - | 1 | - | - | - | 1 |
| *Sporosarcina luteola* | - | 1 | - | - | - | - | 1 |
| *Staphylococcus succinus* | - | - | 2 | 1 | - | - | 3 |
| *Stenostrophomonas maltophilia* | - | - | 1 | - | - | - | 1 |
| *Stenostrophomonas rhizophila* | - | - | 1 | - | - | - | 1 |
| *Streptococcus vestibularis* | - | - | - | - | 1 | - | 1 |
| *Viridibacillus neidei* | 1 | - | - | - | - | - | 1 |
| Total Nr. Of isolates | 123 | 32 | 137 | 122 | 26 | 12 | 452 |

-: zero isolates obtained
