# Supplementary material for: Analysis of seed-associated bacteria and fungi on staple crops using the cultivation and metagenomic approaches
Source: Folia Microbiol (Praha). 2022 Feb 26;67(3):351–61. doi: 10.1007/s12223-022-00958-5 (PMC9072454; doi:10.1007/s12223-022-00958-5)
Supplement: Supplementary file 5 — Supplementary file5 (DOCX 22 KB) [file 12223_2022_958_MOESM5_ESM.docx]

**Table S5 List of all obtained fungal isolates**

| **Isolated fungi** | Wheat | | Barley | | Corn | | Total Nr. of obtained isolates |
| --- | --- | --- | --- | --- | --- | --- | --- |
| **Farming system** | conventional | organic | conventional | organic | conventional | organic |  |
| *Alternaria alternata* | 5 | - | 7 | 6 | - | - | 18 |
| *Alternaria infectoria* | - | - | - | 2 | - | - | 2 |
| *Arthrinium arundinis* | 1 | - | - | - | - | - | 1 |
| *Aspergillus clavatus* | - | - | - | - | 1 | 1 | 2 |
| *Aspergillus flavus* | 2 | 1 | - | 2 | 1 | 1 | 7 |
| *Aspergillus montevidensis* | 1 | - | - | - | - | - | 1 |
| *Aspergillus montevidensis/chevalieri* | 1 | - | - | - | - | - | 1 |
| *Aspergillus penicillioides* | - | 1 | - | - | - | - | 1 |
| *Aspergillus puulaauensis* | - | 1 | - | - | - | - | 1 |
| *Aspergillus sp.* | 8 | 1 | 3 | - | - | - | 12 |
| *Aureobasidium pullulans* | - | - | 1 | 2 | - | - | 3 |
| *Cladosporium cladosporioides* | 1 | - | 1 | 1 | - | - | 3 |
| *Cladosporium cladosporioides/ tenuissium* | - | - | 2 | - | - | - | 2 |
| *Cladosporium tenuissium* | - | - | - | 1 | - | - | 1 |
| *Cladosporium sp.* | 1 | - | - | - | - | - | 1 |
| *Fusarium oxysporum* | - | - | 1 | - | - | - | 1 |
| *Fusarium proliferatum* | - | - | - | 1 | - | - | 1 |
| *Fusarium sp.* | - | - | 3 | 3 | - | 1 | 7 |
| *Fusarium verticillioides* | - | - | - | 1 | - | 2 | 3 |
| *Mucor circinelloides* | - | - | 2 | - | - | - | 2 |
| *Penicillium griseofulvum* | - | - | - | - | - | 1 | 1 |
| *Penicillium glabrum/frequentans* | - | 1 | - | - | - | - | 1 |
| *Penicillium oxalicum* | - | - | 2 | 1 | 1 | 1 | 5 |
| *Penicillium sp.* | - | - | 4 | 3 | - | - | 7 |
| *Rhizopus sp.* | - | - | - | - | 1 | 1 | 2 |
| *Trichoderma citrinoviride* | - | 3 | - | - | - | - | 3 |
| *Trichoderma sp.* | - | 1 | - | - | - | - | 1 |
| *Total Nr of obtained isolates* | 20 | 9 | 26 | 23 | 4 | 8 | 90 |

-: zero isolates obtained
