# Supplementary material for: Analysis of seed-associated bacteria and fungi on staple crops using the cultivation and metagenomic approaches
Source: Folia Microbiol (Praha). 2022 Feb 26;67(3):351–61. doi: 10.1007/s12223-022-00958-5 (PMC9072454; doi:10.1007/s12223-022-00958-5)
Supplement: Supplementary file 6 — Supplementary file6 (DOCX 18 KB) [file 12223_2022_958_MOESM6_ESM.docx]

**Table S6 Overlap of the bacterial and fungal community structure (OTUs) according to farming system**

|  | | | **Overlap of OTUs between organically and conventionally produced seeds** | | **OTUs detected only from organically produced seeds** | | **OTUs detected only from conventionally produced seeds** | |
| --- | --- | --- | --- | --- | --- | --- | --- | --- |
| Microorganism | Plant species | **Total detected OTUs** | **Number of OTUs** | **% of all OTUs** | **Number of OTUs** | **% of all OTUs** | **Number of OTUs** | **% of all OTUs** |
| **Bacteria** | Barley | 198 | 164 | 82.8 | 3 | 1.5 | 31 | 15.7 |
|  | Corn | 16 | 3 | 18.8 | 6 | 37.5 | 7 | 43.8 |
|  | Wheat | 152 | 82 | 54.0 | 4 | 2.6 | 66 | 43.4 |
| **Fungi** | Barley | 125 | 119 | 95.2 | 4 | 3.2 | 2 | 1.6 |
|  | Corn | 41 | 17 | 41.5 | 16 | 39.0 | 8 | 19.5 |
|  | Wheat | 130 | 98 | 75.4 | 7 | 5.4 | 25 | 19.2 |
